# A Novel Respiratory Rate Estimation Algorithm from Photoplethysmogram Using Deep Learning Model

**DOI:** 10.3390/diagnostics14030284

**Published:** 2024-01-28

**Authors:** Wee Jian Chin, Ban-Hoe Kwan, Wei Yin Lim, Yee Kai Tee, Shalini Darmaraju, Haipeng Liu, Choon-Hian Goh

**Affiliations:** 1 Department of Mechatronics and Biomedical Engineering, Lee Kong Chian Faculty of Engineering and Science, Universiti Tunku Abdul Rahman, Kajang 43000, Selangor, Malaysia; chinwj97@1utar.my (W.J.C.); kwanbh@utar.edu.my (B.-H.K.); teeyk@utar.edu.my (Y.K.T.); shalinid@utar.edu.my (S.D.); 2 Centre for Healthcare Science and Technology, Universiti Tunku Abdul Rahman, Kajang 43000, Selangor, Malaysia; 3 Electrical and Computer Systems Engineering, School of Engineering and Advanced Engineering Platform, Monash University Malaysia, Bandar Sunway 47500, Selangor, Malaysia; weiyin.lim@monash.edu; 4 Centre for Intelligent Healthcare, Coventry University, Coventry CV1 5RW, UK; haipeng.liu@coventry.ac.uk

**Keywords:** photoplethysmogram, respiratory rate, deep learning, neural network

## Abstract

Respiratory rate (RR) is a critical vital sign that can provide valuable insights into various medical conditions, including pneumonia. Unfortunately, manual RR counting is often unreliable and discontinuous. Current RR estimation algorithms either lack the necessary accuracy or demand extensive window sizes. In response to these challenges, this study introduces a novel method for continuously estimating RR from photoplethysmogram (PPG) with a reduced window size and lower processing requirements. To evaluate and compare classical and deep learning algorithms, this study leverages the BIDMC and CapnoBase datasets, employing the Respiratory Rate Estimation (RRest) toolbox. The optimal classical techniques combination on the BIDMC datasets achieves a mean absolute error (MAE) of 1.9 breaths/min. Additionally, the developed neural network model utilises convolutional and long short-term memory layers to estimate RR effectively. The best-performing model, with a 50% train–test split and a window size of 7 s, achieves an MAE of 2 breaths/min. Furthermore, compared to other deep learning algorithms with window sizes of 16, 32, and 64 s, this study’s model demonstrates superior performance with a smaller window size. The study suggests that further research into more precise signal processing techniques may enhance RR estimation from PPG signals.

## 1. Introduction

Respiratory rate (RR) is a vital sign that provides basic information about a patient’s respiratory condition and health status. Despite the development of new technical approaches, in clinical practice, RR is still manually counted or estimated using electrocardiogram (ECG) signals [1,2]. However, counting by hand is subjective and prone to mistakes, particularly in patients who are seriously ill or using mechanical breathing [2,3]. ECG signals need a lot of patient probing, which is inappropriate in many situations, including surgery and ambulance transfers [4].

Photoplethysmography (PPG) has gained popularity as a promising non-invasive method to calculate RR in recent years [3]. Photoplethysmography is easily acquired using a straightforward finger probe and measures variations in blood volume in peripheral tissues [5]. Photoplethysmography enables the simultaneous estimation of multiple physiological parameters using a single peripheral sensor, e.g., heart rate variability, oxygen saturation (SpO_2_), blood pressure, and RR [5,6]. Photoplethysmography signals are modulated by respiration in amplitude, baseline, and frequency, enabling RR extraction in different approaches. PPG-based RR estimation has gained user acceptance by reducing the burden of wearing devices with several probes that record ECG to monitor RR for out-of-hospital patients continuously [2]. However, PPG signals are sensitive to noises and are influenced by multiple physiological factors, including age, measurement site, blood pressure, and neural activities [7,8,9]. The challenge towards clinical application lies in the accurate, reliable, and robust estimation of RR from PPG data.

Several algorithms have been developed for RR estimation, each employing distinct techniques to achieve accurate results. Filter-based techniques form one approach, incorporating digital filtering [10,11], low-pass finite impulse response filter [12,13], Butterworth band-pass filter [10,14], and adaptive infinite impulse response notch filter [15,16]. These methods have demonstrated performance with a mean absolute error (MAE) of 2.2 breaths/min, utilising a 5 s window size on the BIDMC datasets. Another approach involves feature-based techniques, encompassing Variable-Frequency Complex Demodulation [10,17], continuous wavelet transforms [10,17,18], Autoregressive Modeling [18,19,20], Beat Detection [21,22], and pulse segmentation [11,21]. State-of-the-art classical algorithms utilising feature-based techniques and time domain RR estimation techniques have achieved an MAE of 2.08 breaths/min with the same 5 s window size. Additionally, feature fusion and selection techniques can be implemented to consolidate the results from different feature-based methods or choose the most suitable technique for each dataset, potentially reducing the MAE to 1.95 breaths/min with a 5 s window size [23].

Numerous features in the signal domain have been extensively employed for RR estimation. However, the investigation into classical methods encounters a bottleneck, as enhancing the performance of RR estimation through signal features proves challenging [23]. Despite several studies employing deep learning algorithms, their performance has yet to surpass that of classical methods. Notably, current deep learning algorithms necessitate large window sizes for optimal performance, indicating a substantial gap in achieving superior results compared to classical methods [24]. Considering these observations, there is a compelling need to delve deeper into exploring deep learning algorithms for RR estimation, aiming to bridge the existing performance gap and potentially surpass the efficacy of classical methodologies.

In addition to that, ongoing research focuses on deep learning neural networks, with notable models like RRWaveNet ResNet, RespWatch, CycleGan and RespNet [24,25,26,27,28]. The state-of-the-art neural network is RRWaveNet, which has pushed the performance even further, achieving an MAE of 1.62 breaths/min and 1.59 breaths/min for BIDMC and CapnoBase Datasets, respectively. However, it utilises a longer 32 and 64 s window size [24]. The RRWaveNet algorithm employs a sophisticated architecture featuring multiple convolutional neural networks (CNNs). Signal data for its training and evaluation are sourced from the BIDMC and CapnoBase datasets through oximeter recordings [24]. This algorithm demonstrates commendable performance in terms of MAE, showcasing its efficacy in accurately estimating respiratory rates. Using diverse datasets enhances its robustness and generalisability, making RRWaveNet a promising and effective model for respiratory rate estimation.

The present study focused on RR estimation from PPG signals. A vital parameter is the window size, which represents the signal duration required for RR estimation. For instance, a 10 s window size provides RR readings after 10 s of applying the PPG sensor, offering real-time estimations with the average RR over the specified window. Shorter window sizes contribute to more immediate results, particularly crucial when a patient’s breathing rate undergoes sudden changes. In contrast, larger window sizes, such as 32 or 64 s, delay the identification of abrupt respiratory rate drops. Given the importance of timely RR monitoring, especially for pulmonary patients at risk of brain damage after 4 min of oxygen deprivation, achieving a low window size becomes paramount in terms of developing an effective RR estimation algorithm [2].

The predominant optimisation of convolutional neural networks (CNNs) characterised algorithms aiming to comprehend signal patterns for RR estimation, with various architectures explored for signal learning and RR estimation. In the current study, a distinctive approach has been taken by introducing a Long Short-Term Memory (LSTM) layer to the architectural configuration [29]. Notably, the preceding studies had yet to investigate the performance of the LSTM layer in this context. As a type of recurrent neural network, the LSTM layer possesses the ability to analyse sequential input, optimising and preserving long-term relationships within the sequence. This is particularly crucial to our study’s objective to reduce the window size. Consequently, our study focuses on exploring a combined neural network, integrating both CNN and LSTM layers, to uncover its potential impact on improving RR estimation accuracy through a nuanced analysis of signal patterns and long-term relationship recognition within the data sequence.

The performance of PPG-based RR estimation can still be improved by reducing the MAE. Additionally, there is potential to decrease the window size for more precise and real-time estimation. The potential for neural networks to improve RR estimation is vast and largely untapped in current research. Numerous neural network architectures and configurations are yet to be explored for this purpose. Moreover, there is room for innovation in reducing the window size for more real-time and responsive estimation, which could significantly enhance performance. The field of RR estimation remains open to a wide array of neural network possibilities, underscoring the need for further exploration and innovation in this domain to achieve more accurate and efficient results.

This study aims to develop an algorithm that can estimate RR accurately and reliably from PPG signals, where the best algorithmic parameters are identified. The performance in different window lengths was assessed to provide a reference for PPG-based real-time measurement of RR.

## 2. Materials and Methods

**Figure 1:**
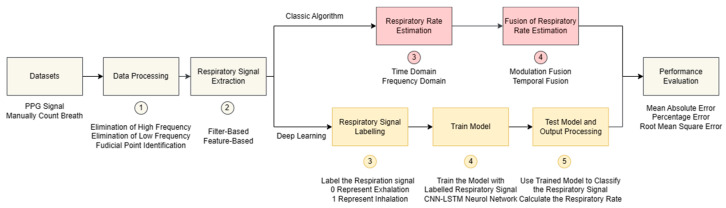
The process of classical algorithm and deep learning algorithm.

In this study, both classical and deep learning algorithms were optimised to estimate RR from PPG signals. A classical algorithm was employed to assess the performance of different classic techniques, determining the optimal combination for RR estimation through rigorous evaluation. Following the performance evaluation of the classical method, a deep learning algorithm was developed to enhance performance compared to previous iterations. The entire process, encompassing the classical and deep learning algorithms, is visually represented in Figure 1, illustrating the roadmap followed in this study for RR estimation.

### 2.1. Source of Data

#### 2.1.1. BIDMC Dataset

The BIDMC datasets are a collection of respiratory signals from intensive care patients’ ECG, PPG, and impedance plethysmography [30,31]. The datasets are meant to be used to analyse RR algorithm performance, representing how well they might work in a real-world critical care setting [30,31].

The primary data for this study were obtained from severely ill patients at the Beth Israel Deaconess Medical Centre in Boston, MA, USA. For each recording in the dataset, individual breaths were meticulously annotated by two individuals using the impedance respiratory signal. The dataset comprises 53 recordings from different patients, each lasting 8 min [32]. These datasets encompass various physiological signals sampled at 125 Hz, including the ECG, impedance respiratory signal, and PPG. Physiological parameters such as heart rate, RR, and SpO_2_ are sampled at 1 Hz. Additionally, the datasets contain manually annotated breaths and fixed parameters like age and gender [21]. The manually annotated breaths were used as reference RR in this study.

#### 2.1.2. CapnoBase Dataset

The CapnoBase dataset comprises 8 min recordings from 42 subjects, including 13 adults and 29 children and neonates, which were obtained during elective surgeries and routine anaesthesia [33]. These recordings include ECG and PPG signals and capnometry signals measuring inhaled and exhaled carbon dioxide (CO_2_), serving as a reference for breathing rate. The CapnoBase TBME RR benchmark dataset provides CO_2_ waveforms (capnogram) and PPG data, along with expert-provided labels for pulse peaks in PPG and breaths in CO_2_ [33]. Researchers can use this benchmark dataset to test and compare algorithms.

### 2.2. Classical Respiratory Rate Estimation Algorithm

#### 2.2.1. Respiratory Rate Estimation Toolbox

The Respiratory Rate Estimation (RRest) toolbox is a MATLAB-based toolbox offering a comprehensive array of algorithms designed for estimating RR from physiological signals [32]. This toolbox is an integral part of the larger RR Estimation project and encompasses a wide range of algorithms that have been documented in prior literature. Rrest is adept at estimating RR using windows of ECG and pulse oximetry (PPG) signals while providing a reference RR derived from concurrent respiratory signals, such as Impedance Pneumography data. It serves as a valuable resource, particularly when researchers wish to compare their novel RR algorithms with established ones [21]. Rrest offers a library of validated algorithms and streamlines the statistical analysis of algorithm performance, facilitating the computation of various statistics commonly used for performance evaluation [32].

#### 2.2.2. Respiratory Signal Extraction

**Figure 2:**
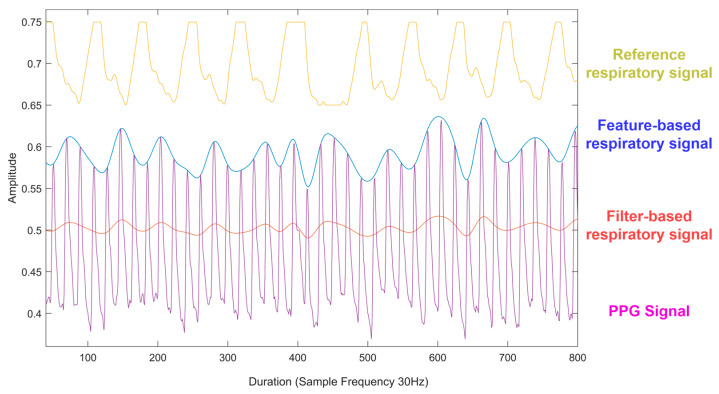
Respiratory signal extraction.

There are two main respiratory signal extraction method categories: feature-based extraction and filter-based extraction. Techniques used in filter-based extraction include wavelet decomposition and band-pass filtering to remove non-respiratory frequencies. A measurement, such as pulse wave amplitude, is taken out of each cardiac cycle as part of feature-based extraction [34] (Figure 2).

The original signal was extracted to produce a respiration signal, which is a time series dominated by breathing modulation. Eliminating extremely low frequencies was the first stage, which was universal to all strategies. A high-pass filter with a 3 dB cutoff frequency of 4 breaths/min was used to achieve this [35]. Both feature-based and filter-based respiratory signal extraction techniques were employed, which dictated the intermediate steps. The next stage, which was the same for all approaches, was employing a band-pass filter with 3 dB thresholds of 4 and 60 breaths/min to exclude frequency unrelated to respiration from the reconstructed signal [36]. The following phases comprised the feature-based extraction process, which involved extracting a temporal sequence of beat-by-beat feature readings. Using low-pass filters with 3 dB thresholds from 100 and 35 Hz, extremely high frequencies were removed for the PPG signal [35].

This algorithm applied adaptive pulse segmentation and artifact detection for PPG. To accomplish automated heart rate and artifact detection, the periodic component of the PPG signal must be removed. The maximal volume peak optimisation of the regular heartbeat pulsations is sometimes followed by a second peak known as the dicrotic notch [37]. A line segmentation method processes the waveform and divides the PPG into pulses. This approach effectively enables the desired trend computation since PPG pulse components are based on a morphological shape that successive lines can identify.

The Iterative-End-Point-Fit and Incremental algorithms that make up the Incremental-Merge Segmentation (IMS) technique were initially created for computer vision and mobile robotics applications [36]. The segmentation-assisted ECG signal compression method has also been applied. The IMS algorithm may be implemented in real time and is simple, quick, and based on a sliding-window structure [37]. The irregularly sampled feature-based respiratory signals are then resampled using linear, interpolation, cubic spline interpolation, or Berger’s technique at a constant sampling rate. Band-pass filtering is an option that can be used after these methods if desired [21].

Linear interpolation is a method for fitting the points to a curve using a linear polynomial, such as the line equation. This is equivalent to drawing a line between two points in a dataset to connect them. The process of fitting the curve by connecting those points with a higher degree polynomial is known as polynomial interpolation. Low-degree polynomials are used in each of the intervals in spline interpolation, which is like polynomial interpolation x’ in that it selects the polynomial parts to fit together smoothly [38]. The outcome is a function known as a spline. Using cubic spline interpolation, finding a curve that connects data points with a degree of three or fewer is possible. Splines are polynomials that have continuous first and second derivatives and are smooth and continuous across a specified plot [38].

#### 2.2.3. Respiratory Rate Estimation

Respiratory Rate Estimation was performed after RR signal extraction using frequency-based techniques and time domain detection. Frequency-based pinpoint the frequency component associated with respiration. Time domain detection includes peak detection on respiratory signal and identifying zero-crossings with a positive gradient along the RR signal. The RR was found to correlate to the frequency of the spectral peak with the largest magnitude from 4 to 60 breaths/min using spectral analysis.

The fusion stage has drawn much attention lately because of the gains in algorithm performance seen when it is used. To combine simultaneous RR estimates for each modulation that were generated using the feature-based methodology, two modulation fusion algorithms were optionally optimised. Smart fusion is the anticipated RR signal’s quality, which is assessed using the BW, AM, and FM respiratory signals. If the standard deviation is less than 4 breaths/min, the RR is calculated as the mean; otherwise, no RR is produced [35].

Spectral peak-conditioned averaging optimises the Welch periodogram, and frequency spectra generated from BW, AM, and FM respiratory signals are fused to provide a mean spectrum. Only spectra that include a specific percentage of their spectral power in a frequency range centred on the frequency corresponding to their maximum spectral power are included. The frequency that corresponds to the mean spectrum’s highest power is thought to be RR [35]. It was possible to employ temporal fusion to smooth out subsequent RR estimations from the same person. It can be used with or without modulation fusion beforehand [35].

### 2.3. Neural Network for Respiratory Rate Estimation

A deep learning algorithm has been developed to estimate RR. BIDMC datasets have been used to develop the deep learning algorithm. The datasets were cut from 53 eight-minute data to 424 one-minute data for training and testing. The algorithm combines a CNN and a LSTM neural network. The convolutional layer was used as the first layer to classify the RR signal. A convolutional neural network can extract features from both spatial and temporal dimensions. A Long Short-Term Memory network is a type of recurrent layer that can improve the prediction ability of the deep learning model. Long Short-Term Memory network operates on sequential data by iterating through time steps and capturing intricate relationships between them, facilitating the learning of long-range dependencies within the sequence. The input of the deep learning model was a 30 Hz RR signal, and the output was the labelling of inhalation and exhalation.

#### 2.3.1. Deep Learning Algorithm

Similar data processing and RR signal extraction were applied before the deep learning algorithm to obtain a clean RR signal. To reduce the training time, the RR signal was resampled from 125 Hz to 30 Hz, which retained the signal’s pattern and reduced the input data’s size. Then, a severe baseline difference was found due to the different blood pressures of the patients. Therefore, the zero-score method was applied to remove the baseline difference and rescale all the RR signals between negative two and positive two.

The labelling process is carried out by cutting the zero as the midline. Any signal higher than zero is considered an inhalation signal and is labelled as one. Meanwhile, signals lower than zero are considered exhalation signals and are labelled as zero. Many RR signals have artifacts and fluctuations. Therefore, the labelling should be modified for the training model to learn all the artifacts and fluctuations. All the train and validation RR signals should be labelled for training.

**Figure 3:**
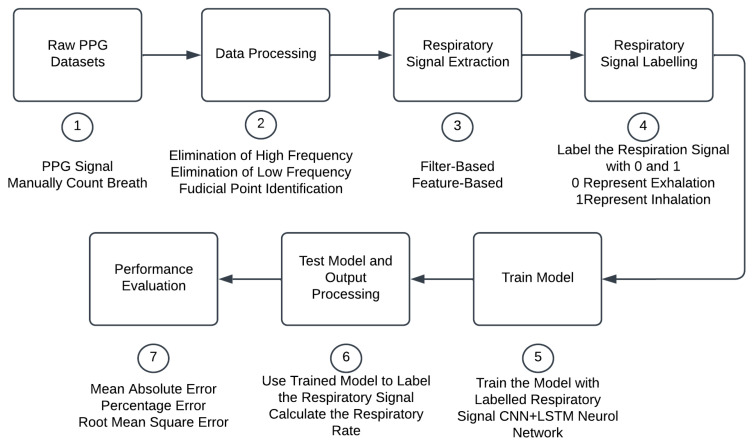
Deep learning algorithm flow chart.

After that, the labelled RR signal will be used as the input to train the deep learning model. The RR signal data were split into three parts: training, validation, and testing. The train and validation splits are 80% train data and 20% validation data. The output of the training model shall classify inhalation and exhalation signals. Next, RR was computed based on the number of inhalation signals detected. The performance of this deep learning algorithm can be evaluated by comparing the computed RR to the reference RR. The performance evaluation method includes MAE, RMSE, and percentage error. The whole process of deep learning is shown in Figure 3. This process was applied similarly to the testing data.

The CapnoBase datasets serve as a crucial testing ground for neural network models initially trained on the BIDMC datasets. This evaluation allows researchers to assess and ascertain the model’s performance when applied to diverse datasets beyond its training environment. The objective is to ensure that the model can demonstrate its efficacy across a range of conditions and with various patient demographics, ultimately verifying its suitability for broader clinical applications.

#### 2.3.2. Deep Learning Neural Network Architecture

**Figure 4:**
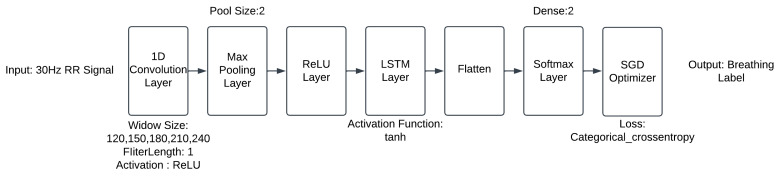
CNN-LSTM neural network architecture.

Clean and scaled 30 Hz RR signals and the labels were fed as input to the CNN-LSTM model. The architecture of the CNN-LSTM neural network is shown in Figure 4.

The neural network takes a 30 Hz labelled RR signal as input. The convolution layer learns from the input signal. Pooling layers offer a technique for reducing the dimensionality of feature maps by condensing information about the presence of features within patches of the feature map. Max pooling will capture the most activated presence of a feature. The Rectified Linear Activation Function, commonly referred to as ReLU, is a mathematical function that behaves in a piecewise linear manner. When the input is positive, it directly outputs the same value; otherwise, it outputs zero. ReLU has gained widespread popularity as the default activation function in various neural network architectures due to its ease of training and its tendency to yield improved performance in many cases.

The model uses LSTM as the recurrent neural network. It is a fundamental building block to analyse sequential input, such as time series or text written in natural language. It can optimise and retain long-term relationships between sequences. The flattened layer is a vital bridge between CNNs and artificial neural networks. It facilitates the seamless integration of CNNs with ANNs, enabling the neural network models to acquire a deep understanding of intricate patterns in data and subsequently make accurate predictions.

The final layer of the mode is the SoftMax layer. It is a significant layer of the neural network. It transforms the prior layer’s raw scores or logits into probability distributions across many classes. This deep learning model gives the label probability on two classes: inhalation and exhalation. Then, the output layer gives the label as 1 (inhalation) or 0 (exhalation). After the classification, the output processing step estimates RR by calculating the number of inhalations and exhalations.

The last part of the model is stochastic gradient descent (SGD) optimisation, a widely used optimisation algorithm used in deep learning. It deals with the classic Gradient Descent methods’ computational inefficiencies while working with huge datasets in deep learning. Utilising SGD, the enormous expense of backpropagation over the whole training set is the driving force behind neural networks in this context. SGD can offset this expense and result in rapid convergence to the optimal.

#### 2.3.3. Deep Learning Neural Network Optimisation Parameters

Several parameters can be modified to optimise the deep learning model performance. The first parameter to optimise is the train–validation and test split. In this study, train–validation data and test data are split by three ratios, which are 20:80, 50:50, and 80:20. Within the train–validation, the train data would be 80%, and the validation data would be 20%. In addition to that, five window sizes were used in the optimisation, which are 120, 150, 180, 210 and 240, corresponding to 4 to 8 s signals, respectively.

In addition, various parameters were optimised to achieve the best model. The CNN layers were tested from one to three, revealing that while additional layers extended training and testing times, they did not significantly enhance performance. Filter lengths ranging from 1 to 5 were examined, with shorter lengths demonstrating better results without substantially increasing processing time because of the downsampling from 125 Hz to 30 Hz in data preprocessing. Pool sizes of 2 to 5 were tested, with larger sizes reducing processing time but adversely impacting performance. Lastly, batch sizes within the range of 5, 10, 15, 20, 25, and 30 were examined, with the determination that the most favourable balance between computational efficiency and optimal performance in respiratory rate estimation was provided by a batch size of 20. This exhaustive exploration across various batch sizes aimed to identify the specific configuration that maximises computational resource utilisation while ensuring excellence in the estimation process.

Training and evaluation were carried out on Intel(R) Core (TM) i5-10300H CPU with 8 GB of RAM hosted by Nvidia GeForce GTX 1650 GPU. In each experimental run, 100 training epochs with a batch size of 20, a pool size of 2, and a filter length of 1 were applied. To mitigate over-fitting, an early stopping technique was implemented during the training phase. The model that achieved the lowest validation loss was preserved and subsequently employed for testing.

### 2.4. Performance Evaluation

Three parameters were used to evaluate the algorithm’s performance. It includes MAE, percentage error, and root mean square error (RMSE). For the performance evaluation, the lower the value, the better the performance in RR estimation. The formula of the performance evaluation parameter is shown in the equation below.

(1) $$MAE=\frac{1}{N}\sum_{i=1}^{N}\left|x_{i}-\hat{x_{i}}\right|\left(breaths/min\right)$$

(2) $$Percentage\;error=\frac{Estimate\;RR-Reference\;RR}{Reference\;RR}\times 100\%$$

(3) $$RMSE=\sqrt{\frac{1}{N}\sum_{i=1}^{N}{\left|x_{i}-\hat{x_{i}}\right|}^{2}}(breaths/min)$$

where $x_{i}$ is a reference value and $\hat{x_{i}}$ is an estimated value of the signal, and N is the total number of samples in the signal.

## 3. Results

### 3.1. RRest Toolbox Algorithm That Utilised Fusion Method

In the 150 combinations that included fusion techniques, the MAE values ranged from 1.95 breaths/min in the best case to 35.06 breaths/min in the worst. The measurements show a percentage error of 10.89%, an RMSE of 2.82 breaths/min, and a CP2 of 66%. These combinations include mean Peak Amplitude as a feature selection technique, breath detection via combined trough and peak detection as the RR estimation techniques and temporal smoothing as fusion techniques (Table 1).

### 3.2. Deep Learning Neural Network Optimisation

The hyperparameter for the CNN-LSTM neural network has been optimised. The final neural network contains a 1-D convolution layer, a max pooling layer, a ReLU layer, an LSTM layer activated by tanh, a flatten layer, a SoftMax layer and an SGD optimiser layer. The optimal model uses a pool size of 2, a filter length of 1, a window size of 210 and a 50% train–test split. The performance of the best model has achieved an MAE of 2.02 breaths/min, a percentage error of 11.82% and an RMSE of 2.6 breaths/min.

**Figure 5:**
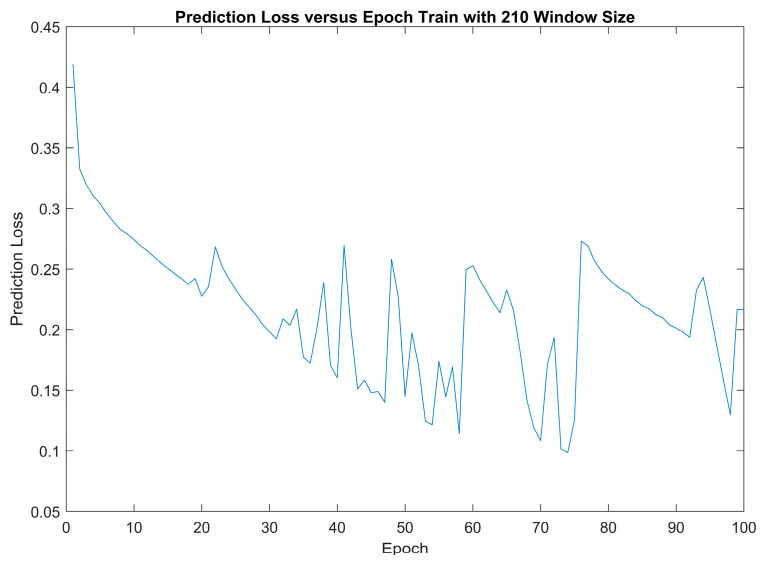
Prediction loss versus epoch train with a 210 window size.

In Figure 5, the chart shows the variation in loss of validation data over the training epochs with 210 data points or 7 s as the window size. It is worth noting that the loss starts to exhibit fluctuations after the 30th epoch, indicating the stabilisation of the model’s performance. To select the most suitable model for testing, consider the model with the lowest loss over the course of 100 epochs. This rigorous methodology led to the identification of an optimal model, achieving an impressively low loss of 0.098; this exceptional performance milestone was reached precisely during the 74th epoch.

#### 3.2.1. Performance of Different Window Sizes

Table 2 illustrates the relationship between window size, performance, and processing time in the context of RR estimation. Notably, it reveals that as the window size increases, performance also improves, reaching an optimum at a 210 window size. However, it is essential to note that this improvement comes at the cost of increased training and test times. A saturation point is observed in the training process when the window size is extended to 210 and further to 240. Consequently, the decision was made to halt the window size expansion at 240, recognising that pushing it beyond this point yielded diminishing returns, likely due to computational constraints or other limiting factors.

The model with the lowest loss over the course of 100 epochs was selected as the model for testing. Therefore, the optimal model was selected during the 74th epoch of a 210 window size, which achieved a loss of 0.098. This underscores the model’s stability and its readiness for thorough testing and evaluation.

#### 3.2.2. Performance of Different Train–Test Split

Interestingly, it was observed that splitting the data into a 50% training and 50% testing configuration produced outcomes comparable to those achieved with an 80% training split. Consequently, opting for a 50% training split streamlines the training process and reduces computational demands, making it a practical choice for model optimisation. This fine calibration of the train–test split parameter is critical for ensuring effective learning and the feasibility of the model development effort. Table 3 shows different train–test split performance on 210 window sizes.

## 4. Discussion

### 4.1. Performance of Classical Algorithm

The initial phase of respiratory rate (RR) signal extraction involves employing either feature-based or filter-based techniques. Feature-based methods encompass key aspects like Amplitude Modulation, Frequency Modulation, Baseline Wander, Peak Amplitude, and Trough Amplitude [23,39]. On the other hand, filter-based techniques encompass band-pass filtering, filtering using the centred-correntropy function, and wavelet extraction [23]. Subsequently, RR estimation is performed on the extracted RR signal utilising both frequency domain techniques (e.g., Fourier transform and auto-regressive spectral analysis) and time domain techniques (e.g., peak detection and Zero-crossing breath detection). Fusion methods, including smart fusion and temporal fusion, play a crucial role in refining the RR signal. Smart fusion adaptively selects from Amplitude Modulation, Frequency Modulation, and Baseline Wander to combine their features for enhanced RR signal estimation [23]. Temporal fusion is employed to smooth out the RR signal from the same individual, ensuring a more stable and reliable respiratory rate estimation.

This study’s comprehensive exploration of respiratory rate estimation using classical techniques involved testing 274 combinations. Out of these, 150 combinations incorporated fusion methods, revealing that using fusion techniques significantly enhanced performance. The evaluation of various classical method combinations allowed for identifying the most effective technique, shedding light on the optimal approaches for accurate RR estimation. This systematic assessment underscores the importance of fusion methods in refining classical techniques and highlights the specific combinations that yield superior performance in RR estimation. Table 1 shows the top 10 best-performing algorithms with the fusion method ranked with MAE value. All top 10 algorithms used the time domain techniques for RR estimation, indicating that the time domain techniques have better performance than frequency domain techniques in RR estimation with the fusion method in this dataset.

### 4.2. Performance Analysis in Deep Learning Model

Compared with window size, it is worth noting that this 7 s window size aligns closely with conventional algorithms, which typically employ a 5 s window. However, it is vital to recognise that the choice of window size impacts both the model’s training and testing times significantly. For instance, a 120-sized window necessitates approximately 14 h for training and a mere 3 min for predictions on 200 min of signal data. In contrast, a 240-sized window extends the training time considerably, requiring roughly 55.5 h, with predictions for the same 200 min of signal data taking approximately 13 min. This underscores the intricate trade-off between window size, training duration, and the responsiveness of the algorithm in real-time scenarios.

In comparison with the train–test split, the allocation of data into training and testing sets is a crucial parameter in model development. Striking the right balance is essential, as having too little data for training can hinder the model’s ability to grasp intricate patterns and may result in missing out on essential scenarios not adequately represented in the training set [25]. Conversely, an overly large training dataset can significantly extend the training time, necessitating substantial computational power and potentially leading to kernel disconnections in platforms like Visual Studio Code and runtime errors in platforms like Google Colab.

### 4.3. Performance Comparison with the State of the Art

Three contemporary algorithms were found to estimate RR. The first algorithm was the RRWaveNet Model, a novel deep learning technique recently published in the field and achieved an MAE of 1.62 with a 32 s window size. The second algorithm was the RRest Toolbox, a powerful tool designed for the comprehensive assessment of classic techniques in RR estimation. Through 274 different combinations of these techniques, the lowest MAE of 1.95 was attained. Lastly, within the scope of this project, an algorithm was developed that combines CNN with LSTM networks. This unique model delivered an MAE of 2.42 under optimal conditions.

However, the testing phase encountered several challenges. As compared to a full signal set of 424 min applied to the RRest Toolbox, only 217 min of signals were utilised during the deep learning testing phase due to the fact that a 50:50 train–test split was applied, which is considerably less than the 424 min used for the RRest Toolbox. Additionally, 12 non-consecutive 1 min signal segments exhibited severe fluctuations, significantly affecting the model’s performance. Strikingly, upon removing these troublesome signal segments, a substantial enhancement in the model’s accuracy was observed, where MAE was reduced from 2.42 to 2.02 with a low window size of 7 s.

To ensure an equitable comparison, identical data preprocessing steps were applied to all datasets and the classic methods were evaluated using the RRest Toolbox. These classical techniques achieved an MAE of 1.90, which held its own in contrast to the outcomes after eliminating fluctuating signal segments. This observation suggests that deep learning algorithms harbour untapped potential for further exploration. In summary, the findings in this study imply that, with meticulous data preprocessing and rigorous signal quality control, deep learning methods can perform on par with well-established classical techniques in this specific domain. Expanding the variety of datasets during the training of deep learning methods has been shown to enhance model performance [25]. The model exhibits improved capabilities when exposed to a more diverse dataset. Additionally, having better computational power enables the incorporation of more extensive datasets, fostering a deeper understanding and refinement of the model. Moreover, using synthetic data becomes feasible when there is sufficient computational power, contributing to the augmentation of the training dataset [25] (Table 4).

To assess the model’s performance across diverse datasets, CapnoBase datasets were employed for testing, maintaining consistent data preprocessing and scaling techniques. The BIDMC dataset focuses on adults, predominantly adults over 50 years of age, in intensive care units. In contrast, the CapnoBase dataset encompasses a variety of ages, including both adults and children undergoing elective surgery and anesthesia. Both datasets are inclusive of both male and female patients. The CapnoBase dataset, with its broader representation of patient demographics and medical contexts, provides a more comprehensive testing ground for model robustness compared to the BIDMC dataset, which focuses more on a specific age group.

Remarkably, the model demonstrated effective RR estimation with a commendable MAE of 1.99 breaths/min. Further refinement was achieved by removing segments with severe signal fluctuations to mitigate artifacts, resulting in an even more impressive MAE of 1.24 breaths/min. The MAE achieved by this model exceeded the performance of the state-of-the-art deep learning algorithm, which is 1.59 breaths/min. This model exhibits several strengths, including its ability to operate with low window size, minimal processing time, and adaptability to perform well when applied to other datasets, underscoring its robustness and suitability for various applications (Table 5).

When comparing various deep learning algorithms, it becomes evident that window size and MAE are pivotal parameters signifying algorithm performance, with improvements noted as more studies emerge. While increasing the window size tends to result in a better MAE, the significance of a smaller window size for rapid responsiveness to changes in RR cannot be understated. Despite RRWaveNet’s higher MAE, optimal performance requires a larger window size, only achieving lower MAE values when using 32 and 64 s windows [24]. In this study, although the MAE is slightly higher than other deep learning algorithms, the window size is remarkably reduced to only 7 s, providing efficient RR estimation within a shorter timeframe for faster response. Balancing window size and MAE poses a significant challenge in algorithm development, often requiring trade-offs. The current study incrementally improves window size and MAE, recognising the ongoing opportunity for exploration in uninvestigated neural network layers and architectures with the potential for enhanced algorithm performance.

### 4.4. Fluctuation Signal Elimination

**Figure 6:**
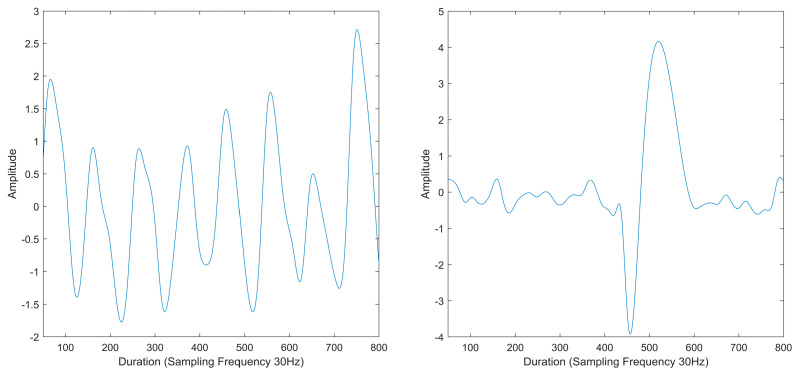
Normal signal (**Left**); fluctuation signal (**Right**).

Eliminating fluctuation signals is crucial for ensuring the accuracy of respiratory rate (RR) estimation, as motion artifacts, light disruptions, and noise can adversely affect signal quality, leading to the emergence of fluctuation signals. In the BIDMC datasets, 12 one-minute signal segments with severe fluctuation signals were identified and removed due to their potential to introduce extremely large errors. Such errors could result in a reported RR of 30 breaths/min, while the reference RR was only 14 breaths/min. To attain a more precise estimation, it is imperative to eliminate fluctuation signals that may arise during instances of motion artifacts, light disruptions, or noise, thereby enhancing the overall accuracy of RR measurements. Figure 6 shows the comparison between the normal signal and the fluctuation signal.

### 4.5. Clinical Application, Limitations, and Future Directions

#### 4.5.1. Clinical Application

The clinical application of RR estimation from PPG signals, as demonstrated by the findings in this study, holds promising implications for patient monitoring in various medical conditions [40]. A single-probe oximeter recording PPG signals proves to be an efficient tool for long-term patient condition monitoring, offering a distinct advantage over ECG, which typically requires multiple probes. The PPG-based device’s simplicity, portability, and user-friendly nature make it a practical choice in clinical settings.

In a clinical setting, RR estimation burdens patients and healthcare providers, often requiring multiple probes from ECG or manual recording and monitoring, particularly in ambulatory services. However, implementing an accurate, fast-response, and low-processing requirement RR estimation algorithm into a bedside monitor and a simple oximeter offers a transformative solution [1]. With this approach, all RR data can be easily recorded and monitored, streamlining the process and significantly reducing the time and effort involved [1]. Such an algorithm enables the swift identification of abnormal RR, facilitating prompt responses and interventions when needed, ultimately improving the efficiency and effectiveness of RR monitoring in clinical scenarios.

For chronic pulmonary diseases such as Chronic Obstructive Pulmonary Disease and obstructive sleep apnea, continuous and reliable RR monitoring is crucial for disease management and early detection of exacerbation. In these cases, the low window size and real-time responsiveness of the deep learning model in the study can significantly contribute to effective patient care. In acute pulmonary diseases like acute respiratory distress syndrome or pneumonia, rapid and accurate monitoring of RR is vital for timely intervention. The fast and reliable response the PPG-based device provides with the developed algorithm becomes particularly critical in such acute scenarios, ensuring the prompt detection of RR changes.

#### 4.5.2. Limitations

In this study, limitations exist in the domain of RR estimation from PPG signals, with computational constraints being a notable challenge. Model training and development are hindered by limitations in computational power, often resulting in interruptions such as kernel disconnections that halt the training process. With improved computational capabilities, model training could be more robust and efficient, allowing for the inclusion of more extensive datasets and achieving better performance with reduced errors.

Another limitation arises from the availability of open-source datasets, which may not be sufficiently comprehensive to fully develop and refine the algorithm. The need for a wider variety of datasets is evident, as a more diverse range of data is essential for enhancing the algorithm’s generalisation and overall accuracy. In essence, overcoming these limitations hinges on advancements in computational resources and expanding diverse datasets to foster the continual improvement and applicability of respiratory rate estimation algorithms based on PPG signals.

#### 4.5.3. Future Directions

Guiding the future progression of the project for respiratory rate estimation based on PPG signals involves strategic considerations. Addressing computational constraints by exploring enhanced resources or refined algorithms is a pivotal focus for advancing model training. This may encompass utilising advanced hardware, cloud-based computing, or implementing streamlined parallel processing methods to surmount the existing limitations.

Future advancements in artifact identification and reconstruction are essential for refining signal quality and optimising RR estimation performance. To address the impact of artifacts on signal accuracy, innovative approaches may involve advanced signal processing and dedicated deep learning methods for effective artifact detection and reconstruction. Developing robust algorithms capable of distinguishing PPG signals from artifacts and efficient reconstruction techniques is critical for enhancing RR estimation.

Equally essential is the expansion of the dataset repository. Establishing partnerships with healthcare institutions or gaining access to more diverse datasets is imperative for comprehensive algorithmic development. This approach fortifies the model’s resilience and adaptability, ensuring its efficacy across diverse patient profiles and health conditions.

Furthermore, continual enhancement of the algorithm, particularly in terms of real-time responsiveness and reduced window size, is critical for practical applicability. Integrating more sophisticated signal processing techniques or exploring emerging technologies can provide avenues to further optimise the algorithm’s efficacy.

## 5. Conclusions

In conclusion, the project’s goal of creating a reliable algorithm for accurately estimating RRs from PPG was achieved through a series of well-defined objectives. These objectives encompassed fine-tuning algorithmic parameters, validating accuracy using diverse PPG datasets collected under varying physiological and environmental conditions, enhancing robustness against disturbances such as noise and motion artifacts, and optimising real-time processing to ensure swift and precise RR predictions. This project’s results are of utmost significance for healthcare and monitoring applications, where the accurate estimation of RRs is pivotal in patient care and assessment. In evaluating classical methods, the performance was accurately assessed with 274 different technique combinations. The optimal algorithm emerged, featuring mean Peak Amplitude as the chosen feature selection method, combined trough and peak detection for RR estimation, and temporal smoothing as the fusion technique. Remarkably, this configuration achieved an impressive MAE of 1.9.

A CNN + LSTM neural network was also successfully developed, yielding a slightly higher MAE of 2.02. The model can also be applied to other datasets. The model has been applied to CapnoBase datasets and achieved an MAE of 1.24. This verified the model performance on new PPG raw data. These results underline the comprehensive nature of our project and its ability to excel in both classical and advanced techniques, reinforcing the significance of our findings for healthcare and monitoring applications where precise RR estimation is paramount.

## Figures and Tables

**Table 1:** Top 10 technique combination performance with fusion.

Rank	Combination	MAE	Percentage Error	RMSE
1	Peak Amplitude, Trough and Peak Detection, Temporal Fusion	1.95	10.89	2.82
2	Peak Amplitude, Positive Gradient Zero-Crossing Detection, Temporal Fusion	2.03	11.35	2.88
3	Trough and Peak Detection, Smart Fusion, Temporal Fusion	2.04	11.38	2.62
4	Consecutive Trough Mean Value, Trough and Peak Detection, Temporal Fusion	2.04	11.40	2.88
5	Consecutive Trough Mean Value, Positive Gradient Zero-Crossing Detection, Temporal Fusion	2.04	11.43	2.89
6	Peak Amplitude, Positive Gradient Zero-Crossing Detection, Temporal Fusion	2.10	11.77	3.35
7	Peak Amplitude, Trough and Peak Detection, Temporal Fusion	2.13	11.90	3.39
8	Consecutive Trough Mean Value, Detrend and Detect Trough and Peak, Temporal Fusion	2.15	12.00	3.11
9	Consecutive Trough Mean Value, Spikes and Drop Detection, Temporal Fusion	2.16	12.08	2.87
10	Positive Gradient Zero-Crossing Detection, Smart Fusion, Temporal Fusion	2.21	12.37	2.87

MAE—mean absolute error. RMSE—root mean square error.

**Table 2:** Performance of different window sizes.

Window Size	MAE	Percentage Error (%)	Training Time (50%)	Testing Time (50%)
120 (4 s)	3.94	23.9	14 h	3 min
150 (5 s)	3.45	20.39	20 h	5 min
180 (6 s)	2.76	16.16	25 h	7 min
210 (7 s)	2.42	14.42	36 h	9 min
240 (8 s)	2.49	15.09	55.5 h	13 min

MAE—mean absolute error.

**Table 3:** Performance of different train–test split.

	Training		Testing	
Train:Test	MAE	Time	MAE	Time
20:80	1.34	16 h	4.27	3 min
50:50	1.3	36 h	2.42	9 min
80:20	1.47	52 h	2.57	12 min

MAE—mean absolute error.

**Table 4:** Performance of different algorithms on the BIDMC dataset.

Study	Method	Window Size (Seconds)	MAE	Processing Time
[26]	RespNet	16, 32, 64	2.45, 2.07, 2.06	-
[24]	RRWaveNet	16, 32, 64	1.87, 1.62, 1.66	-
[25]	ResNet	16, 32, 64	2.25, 2.46, 2.16	-
[27]	RespWatch	16, 32, 64	1.88, 1.96, 1.66	-
[28]	CycleGan	32	1.9	-
This Study	RRest Toolbox	5	1.95	2.5 h
This Study	RRest Toolbox (Removed)	5	1.90	1.5 h
This Study	CNN-LSTM Model	7	2.42	36 h, 9 min (Train, Test)
This Study	CNN-LSTM Model (Removed)	7	2.02	36 h, 9 min (Train, Test)

MAE—mean absolute error. RRest Toolbox (Removed)—Fluctuation signals are removed before applying to the RRest Toolbox. CNN-LSTM Model (Removed)—Fluctuation signals are removed before applying to the CNN-LSTM Model.

**Table 5:** Performance of different algorithms on CapnoBase datasets.

Study	Method	Window Size (Seconds)	MAE	Processing Time
[26]	RespNet	16, 32, 64	5.4, 4.86, 4.48	-
[24]	RRWaveNet	16, 32, 64	1.79, 1.86, 1.59	-
[25]	ResNet	16, 32, 64	2.62, 2.36, 2.29	-
[27]	RespWatch	16, 32, 64	1.87, 2.09, 1.82	-
This Study	RRest Toolbox	5	0.93	2.5 h
This Study	RRest Toolbox (Removed)	5	0.90	1.5 h
This Study	CNN-LSTM Model	7	1.99	7 min (Test)
This Study	CNN-LSTM Model (Removed)	7	1.24	7 min (Test)

MAE—mean absolute error. RRest Toolbox (Removed)—fluctuation signals are removed before applying to the RRest Toolbox. CNN-LSTM Model (Removed)—fluctuation signals are removed before applying to the CNN-LSTM Model.

## Data Availability

Publicly available datasets were analyzed in this study.
